# Cross-Cultural Adaptation of a Health-Related Quality-of-Life Questionnaire for Children with Obstructive Sleep Disorders: Spanish Version of the OSD-6

**DOI:** 10.3390/jcm14051709

**Published:** 2025-03-03

**Authors:** Ignacio Boira, José Norberto Sancho-Chust, Violeta Esteban, Esther Pastor, Miguel Ángel Martínez-García, Eusebi Chiner

**Affiliations:** 1 Pneumology Department, San Juan de Alicante University Hospital, 03550 Alicante, Spain; 2 Pneumology Department, La Fe University and Polytechnic Hospital, 46026 Valencia, Spain

**Keywords:** sleep disorders, adenotonsillar hypertrophy, OSD-6, psychometric properties

## Abstract

**Background/Objectives:** To translate the OSD-6 questionnaire (6-item quality of life questionnaire for children with obstructive sleep disorders) into Spanish and to assess its psychometric properties and clinical usefulness. **Methods:** We included children with obstructive sleep apnea (OSA). All underwent polysomnography before and after adenotonsillectomy. Study variables included age, sex, symptoms, polysomnography values, body measurements, and Mallampati and Brodsky classification. Parents or caregivers completed the OSD-6 at baseline and 3 to 6 months after adenotonsillectomy. Following translation and back-translation of the instrument, we evaluated its internal consistency, reliability, construct validity, concurrent validity, predictive validity, and sensitivity to change. **Results:** We included 45 boys and 15 girls. Mean body mass index was 18 (standard deviation [SD] 4) kg/m^2^ and mean neck circumference was 28 (SD 5) cm. Seven per cent of children had Brodsky grade 0, 12% had grade 1, 27% had grade 2, 45% had grade 3, and 6% had grade 4. Mean apnea-hypopnea index (AHI) was 12 (SD 7) h^−1^ before adenotonsillectomy. The overall Cronbach’s alpha was 0.8. We found significant concurrent validity in each questionnaire domain and in the overall score. Predictive validity was significant for Mallampati scores (ANOVA *p* = 0.011) and borderline significant for AHI levels (ANOVA *p* = 0.069). The study demonstrated excellent sensitivity to change, both in terms of the overall analysis (*p* < 0.001) and in each domain (*p* < 0.001). Moreover, the test-retest reliability was found to be equally excellent (global intraclass correlation coefficient = 0.92 [0.89–0.95]). **Conclusions:** OSD-6 is a valid and reliable instrument to measure quality of life in children with OSA and can be used in Spanish-speaking countries.

## 1. Introduction

Obstructive sleep apnea (OSA) has a prevalence of 4% in children aged 2 to 6 years. The main risk factors in this age group include tonsillar and adenoid hypertrophy, obesity, craniofacial malformations, neurological and neuromuscular diseases, and gastroesophageal reflux [1]. These conditions usually result in narrowed airways and reduced airflow during the night, leading to sleep fragmentation, intrathoracic pressure changes, and intermittent hypoxia that predisposes to a proinflammatory state, with hypercoagulability, increased sympathetic activity, and oxidative stress [2].

The main long-term consequences of OSA in children are behavioral, neurocognitive, cardiovascular, and metabolic alterations [3,4,5]. Furthermore, OSA negatively influences quality of life in children, modifying their sense of well-being and affecting their psychosocial environment and is related with the development of migraine [6,7]. Validated health-related quality of life (HRQoL) questionnaires are essential tools in clinical practice and research. They focus on the patient’s perspective of the impact of disease or treatment on quality of life. They also provide a standardised way of assessing quality of life, help in clinical decision making and can monitor changes in quality of life after the introduction of a treatment or intervention [8]. There is a need for specific validated questionnaires that are highly sensitive and capable of assessing the efficacy of interventions used to improve HRQoL in children with OSA. Health professionals tend to use generic questionnaires such as the Glasgow Children’s Benefit Inventory [9] or the Youth Quality of Life Instrument [10] in this population.

To limit symptoms associated with OSA, it is crucial to establish an early diagnosis based on several specific and generic factors. The process should begin with exhaustive history taking, including specific questionnaires, followed by a physical examination and diagnostic testing. Polysomnography is the gold standard test for diagnosing OSA [1]. Research has also found home respiratory polygraphy to be useful for diagnosis in children and in clinical decision-making [11].

Questionnaires designed specifically for children and adolescents are relatively recent and have distinct features compared to adult questionnaires. For these reasons, a thorough evaluation of their psychometric properties is warranted. In the OSA setting, existing questionnaires measure HRQoL through parents and caregivers.

Because all were created in English-speaking settings, we have to translate and adapt them to our culture before applying them in clinical practice. This process involves several steps: checking whether the construct to be measured exists in the target culture, translating the items, and evaluating the metric and psychometric properties (validity and reliability) of the new version [12].

Currently, there are few questionnaires designed specifically for childhood OSA. The OSA-18 (created in 2000 and subsequently adapted to and validated in Spanish) is a reliable and sensitive instrument for assessing post-treatment changes in children with this condition [13,14]. Another relevant questionnaire is the Pediatric Throat Disorders Outcome Test (T14), which evaluates the results of treatment in children with OSA and recurrent tonsillitis [15].

The OSD-6, created in 2000 by de Serres et al. [16], is a simple and quick to complete questionnaire for assessing the impact of OSA on quality of life that includes 6 domains that reflect the child’s functioning: physical suffering, sleep disturbance, speech or swallowing problems, emotional distress, activity limitations, and caregiver concerns related to the OSA or associated symptoms. Each domain includes a question designed to measure the overall effect of a group of OSA-related symptoms in each child. (Figure S1). In contrast to the OSA-18 questionnaire, it assesses speech and swallowing problems, and in contrast to the T14 questionnaire, it assesses caregiver concern and emotional impact.

The primary objective of our study was to adapt the original (English) version of the OSD-6 to the Spanish-speaking context. Our secondary objective was to analyze the reliability and validity of the Spanish version in the pre- and post-treatment evaluation of children with OSA.

## 2. Materials and Methods

To adapt the OSD-6, we followed International Test Commission guidelines [17] and proposed guidelines for the cross-cultural adaptation of HRQoL questionnaires [18]. The adaptation process included professional translation and back-translation and a pilot study in patients with different OSA severity [19].

### 2.1. First Phase: Translation to Spanish

The original questionnaire was translated by 2 independent native Spanish translators with the authors’ consent. One of the translators was aware of the concepts of questionnaire and the other was unaware of the objective of the questionnaire. The translators and research group then discussed the resulting texts to obtain a single unified version. This questionnaire was given to parents/caregivers to check it was understandable. A native English translator then back-translated it to ensure accuracy and consistency, and we made any necessary corrections to the Spanish version. The translators assigned a score for each item to reflect the difficulty of translation and back-translation, with 1 representing minimum difficulty and 10 representing maximum difficulty. We also rated the fluency/grammatical correctness of each item of the Spanish version on a scale of 1 to 10. Finally, we graded the conceptual equivalence after the back-translation as follows: A (full equivalence), B (reasonable equivalence, a few dubious expressions), C (dubious equivalence). The present version was distributed to the parents of 10 children, aged between two and 14 years, who had been diagnosed with obstructive sleep apnea (OSA) on the basis of a polysomnography (PSG) study, in order to reach a consensus on the final questionnaire. (Figure S2).

### 2.2. Second Phase: Questionnaire Validation

In the second phase of the study, the psychometric properties of the translated questionnaire were assessed in order to determine whether it exhibited equivalent properties to the original version. Reliability, or internal consistency, was evaluated using Cronbach’s alpha for each scale. The mean correlation coefficient of each domain with the total score from the scale and with the total number of domains was measured. We considered between-group values above 0.4 and individual values (measuring a single dimension) greater than or equal to 0.8 to be adequate. In order to assess the cohesion of the instrument, the correlation between subscales was calculated, as well as the correlation between each subscale and the total. To analyse concurrent validity (construct), Pearson or Spearman correlation values were used (depending on whether the variables followed a normal or non-normal distribution) of the different questionnaire domains, both globally and by subscales.

Convergent validity correlated each item with its domain, and divergent validity correlated each item with other domains. To analyze predictive validity, we compared the groups of patients with different Mallampati scores and different levels of OSA severity according to a set apnea-hypopnea index (AHI) cut-off, through an analysis of variance (ANOVA). To evaluate test-retest reliability, we calculated the intraclass correlation coefficient (ICC) between scores at baseline and after 1 week, for each domain and for the whole questionnaire, administered in the same conditions. We considered ICC values above 0.71 represented good agreement and values between 0.51 and 0.70 represented moderate agreement.

We performed a descriptive analysis of the different variables, evaluating their distribution to select the correct parametric or nonparametric statistical test. Furthermore, an evaluation of the ceiling effect (percentage of responses with the maximum score) and floor effect (percentage with minimum score) was conducted. In addition to these, the percentage of items with no response and the percentage of non-applicable items were also determined.

### 2.3. Third Phase: Sensitivity to Change

In this phase, we assessed changes in HRQoL as a result of treatment, comparing the values of all OSD-6 domains before and after treatment with a repeated measures Student *t* test.

#### 2.3.1. Population

Inclusion criteria: age 2 to 14 years; referral to sleep unit; polysomnography-based diagnosis of OSA.

Exclusion criteria: inability to complete follow-up due to place of residence; immunosuppression (severe malnutrition, primary immunodeficiency, and acquired immunodeficiency syndrome [AIDS]); parent refusal to participate in study; previous airway surgery or other interventions.

#### 2.3.2. Diagnostic Protocol

The diagnostic protocol comprised the following steps: the collection of body measurements (weight, height, body mass index [BMI], BMI percentile, neck circumference); the administration of the data collection questionnaire; the obtention of the Mallampati score [20] and Brodsky grade of tonsil size (grade 0: tonsils in the fossa, grade 1: tonsils outside of the fossa and occupy ≤25% of the oropharyngeal width, grade 2: tonsils occupy 26–50% of the oropharyngeal width, grade 3: tonsils occupy 51–75% of the oropharyngeal width and grade 4: tonsils occupy >75% of the oropharyngeal width); overnight polysomnography in hospital; and the baseline evaluation of the OSD-6 questionnaires administered to parents or caregivers on the day of the test. After polysomnography, pediatric patients suitable for surgical treatment were referred to the otorhinolaryngology department. We repeated the polysomnography 3 to 6 months after the surgery to assess post-treatment changes in respiratory parameters. On the same day, we collected body measurements and symptoms, and readministered the OSD-6.

#### 2.3.3. Polysomnography

To establish a diagnosis, we used the Alice 5 polysomnography system (Phillips Respironics, Murrysville, PA, USA), which records electroencephalogram, electrooculogram, nasal flow (pressure-based sensor), chin electromyography (EMG), chest and abdomen movements, electrocardiogram, and EMG of both legs. We applied the criteria listed in the American Academy of Sleep Medicine to define respiratory events [21]. The sleep technologist interpreted and manually corrected the polygraphic records. We classified OSA severity according to the AHI, as follows: mild OSA, AHI below 5/h^−1^; moderate OSA, AHI of 5–10 h^−1^; severe OSA, AHI above10/h^−1^.

#### 2.3.4. OSD-6 Questionnaire

The OSD-6 includes 35 items grouped into 6 domains: physical suffering (9 items), sleep disturbance (6 items), speech or swallowing problems (6 items), emotional distress (8 items), activity limitation (5 items) and caregiver concern (1 item). Parents rate the domains on a problem scale of 0 (none) to 6 (couldn’t be worse) to reflect how the symptoms affect their child. The total score is the sum of the scores for each domain divided by 6 (number of domains). Possible scores range from 0 to 36, and lower scores reflect a better situation.

#### 2.3.5. Sample Size Calculation

With a confidence level (1-α) of 95%, 3% precision (d), a 5% approximate value of the parameter, a power of 80% and an expected proportion of losses (R) of 15%, we adjusted the sample size to 60 children. We estimated that we would need 20 children to determine test-retest reliability and sensitivity to change. The criterion used to estimate the sample size is based on the statistical requirements for calculating the reliability coefficient, assuming an expected value greater than or equal to 0.85, with that sample and a confidence interval of 95% for Z_α/2_ = 1.96 [22].

#### 2.3.6. Statistical Analysis

We performed a descriptive analysis of the quantitative variables, which we expressed as mean and standard deviation (SD) or median and range, according to the type of distribution as determined by the Kolmogorov-Smirnov test. As well as the calculations used to validate the questionnaires, we used repeated measures ANOVA (normal distribution) or the Kruskal-Wallis (not normal distribution) and multiple comparisons of means tests to assess the changes in scores for each of the domains. Statistical significance was set at *p* < 0.05. We used SPSS version 18 for the statistical analysis.

#### 2.3.7. Ethical Aspects

Parents or caregivers had to give their informed consent to participate in the study. Our project complied with the fundamental principles established in the Declaration of Helsinki, the Council of Europe Convention on Human Rights and Biomedicine, and the UNESCO Universal Declaration on the Human Genome and Human Rights; as well as the requirements established in Spanish legislation in the field of biomedical research, personal data protection, and bioethics. The research ethics committee of San Juan de Alicante University Hospital approved our study (ref. 13/311). The Spanish Society of Pulmonology and Thoracic Surgery (SEPAR) funded this study through a scholarship (ref. 025/2014).

## 3. Results

We included 60 consecutive patients (45 boys and 15 girls) with a mean age of 6 (SD 3) years, a mean BMI of 18 (SD 4) kg/m^2^, and a mean neck circumference of 28 (SD 5) cm. Seven per cent of the sample had Brodsky grade 0, 12% had grade 1, 27% had grade 2, 45% had grade 3, and 6% had grade 4. The mean AHI established through polysomnography before treatment was 12 (SD 7) h^−1^. All patients underwent adenotonsillectomy.

The rating for difficulty of translation assigned by the translators was above 5 for 8 items (23%). After the back-translation, 4 items (11%) were rated reasonably equivalent (B), and 1 (3%) as having dubious equivalence (C). The research group and the translators discussed these equivalence issues (B and C) and the items considered equivalent but with unnatural or grammatically incorrect wording (Table 1).

We reached a final agreed wording for each of these items, so that all items and domains of the questionnaire achieved a score above 7/10 for fluency and correctness. The administration of this new version was conducted on the parents of 10 children, ranging in age from 2 to 14 years, who had been referred to the sleep unit and had received a polysomnography-based diagnosis of OSA. In this way, we reached a consensus on the final version of the questionnaire (Figure S2).

In the analysis of internal consistency or reliability, the Cronbach’s alpha value of the overall questionnaire was 0.8 (excellent), with normal (or almost normal) distributions of the domains (strongest internal consistency: physical suffering, emotional distress, activity limitations and weakest internal consistency: sleep disturbance, speech or swallowing problems and caregiver concerns). Cronbach’s alpha remained stable (0.76 to 0.78) upon removal of each domain. Internal consistency was good, although 20% of respondents selected the highest score for domains 1, 2, and 6, and 15% selected the lowest score for domains 3 and 5. In the overall questionnaire, the ceiling and floor effects were below 2%. Most correlations between items were significant (Table 2).

When analyzing construct validity, as the questionnaire has 6 non-composite domains, we could not apply a factorial analysis to determine the similarity of the construct to the original. Concurrent validity remained stable and showed a positive correlation between all the domains (from 0.23 to 0.77), with a high correlation between the score of each domain and the overall score (from 0.65 to 0.78). We observed the lowest correlations between sleep disturbance and emotional distress (0.23), between sleep disturbance and activity limitations (0.24), and between caregiver concerns and activity limitations (0.27). Table 3 presents the full results.

The questionnaire showed adequate predictive validity for distinguishing between Mallampati scores (ANOVA *p* = 0.011), while the association between OSA-6 scores and AHI levels was borderline significant (ANOVA *p* = 0.069), as shown in Table 4.

Test-retest reliability was also excellent, for the whole questionnaire and for each domain (Table 5). Test-retest reliability showed similar results in the analysis by stratified AHI (no OSA (AHI < 1/h), mild (AHI: 1–5/h), moderate (AHI: 5–10/h) and severe (AHI >10/h)) and according to age (children (from 2 years to 6 years), middle childhood (from 6 to 12 years) and teens (from 12 to 14 years)).

Table 6 presents the results for sensitivity to change, which was excellent in the overall score (*p* < 0.001) and in each domain (*p* < 0.001).

## 4. Discussion

The concept of health in children and young people covers not only physical, psychological and social aspects, but also their ability to carry out age-appropriate activities. The dimensions usually considered are related to daily activities (mobility and self-care), cognitive abilities (memory, concentration and learning), emotions (positive and negative), self-perception, interpersonal relationships (with friends and family) and interaction with their environment (family cohesion, social support). Most HRQoL instruments for children use the psychometric model based on the individual’s ability to discriminate between stimuli of different intensities, with responses collected on scales (typically Likert scales).

Some sleep units use the University of Michigan Pediatric Sleep Questionnaire to screen children with suspected OSA, to rule out the need for polysomnography, or to identify symptoms [23,24]. However, the validated Spanish version seems to differ from the original in the questions related to daytime behavior and sleepiness, aspects that are undoubtedly very important in childhood OSA [25]. The OSD-6 has been validated in Greek [26] and Brazilian Portuguese [27]. It has also been used in a series of patients in India [28], although there is no evidence of prior validation. Similarly, in 2006, Diez-Montiel et al. [29] presented a series of children who had been evaluated with an unvalidated Spanish version of the OSD-6.

Our adaptation of the OSD-6 has a highly psychometric performance as the original version with excellent internal consistency. The predictive validity is adequate, concurrent validity is moderate (though would be improved by a more balanced distribution of OSA severity, as there was a tendency to severe OSA in our sample), and sensitivity to post-treatment changes is excellent. It is essential to have the Spanish version of the questionnaire in order to assess the impact on the quality of life of the disease, select those patients who require surgical treatment and evaluate the improvement in quality of life after surgery.

Our study has some limitations. Because most of the children who come to our sleep unit have been previously assessed by an otorhinolaryngologist or pediatrician, the probability of severe OSA in our participants was high, and severe cases were overrepresented in the sample [30]. In daily practice, the severity distribution would normalize over time. Similarly, it is difficult to find children without clinically significant OSA owing to initial sampling bias with a high pretest probability of having the disease. A further limitation is the fact that validation has not been carried out in other Spanish-speaking countries. Multicenter studies in other Spanish-speaking countries are recommended.

The main strength of our study was that we followed a systematic validation process set out in international guidelines, obtaining a Spanish version of the questionnaire that is equivalent to the original, in a sample of children who underwent polysomnography (considered the gold standard) and who were managed according to a standardized diagnostic and therapeutic protocol.

## 5. Conclusions

Our validation process suggests that the Spanish OSD-6 preserves psychometric properties that are comparable to those of the original version. OSD-6 is a valid and reliable instrument to measure HRQoL in children with OSA. It has clinical utility for pre- and post-treatment evaluation, shows moderate discriminative capacity for severity level, and can provide important information on the impact of childhood OSA through parents or caregivers.

## Figures and Tables

**Table 1:** First phase of questionnaire adaptation.

	Difficulty of Translation	Difficulty of Back-Translation	Conceptual Equivalence	Fluency/Correctness
**Physical suffering**	**1**	**1.5**	**A**	**9**
Sore throat	2	3	A	9
Dry throat	2	2.5	A	8
Nasal congestion	1	2.5	A	8.5
Completely blocked nose	4	5.5	B	7
Bedwetting	7	4	A	7.5
Excessive daytime tiredness	2	3	A	8.5
Failure to gain weight	2	2	A	8.5
Bad breath	4	3	A	9
Overall, how much has physical suffering been a problem for your child during the past 4 weeks because of enlarged tonsils and adenoids?	6	4.5	A	8.5
**Sleep disturbance**	**1**	**1**	**A**	**9**
Snoring	1	1.5	A	9.5
Choking/gasping for air	4	3.5	B	7
Stopping breathing for a few seconds	3	2.5	A	7.5
Restless sleep	2	2	A	9
Difficult to awaken from sleep	3	3	A	7
Overall, how much has sleep disturbance been a problem for your child during the past 4 weeks because of enlarged tonsils and adenoids?	6	4.5	A	9
**Speech or swallowing problems**	**3**	**2.5**	**A**	**9**
Difficulty swallowing certain foods	2	3	A	8
Choking on foods	3	3.5	A	8
Muffled speech	4	3.5	A	9
Nasal sounding speech	4	3.5	A	9
Poor pronunciation	4	4.5	B	7
Overall, how much has speech or swallowing problems been a problem for your child during the past 4 weeks because of enlarged tonsils and adenoids?	6	4.5	A	9
**Emotional distress**	**3**	**3.5**	**A**	**8**
Irritable	1	3	A	8
Frustrated	2	3	A	8
Sad	6	3.5	A	9
Restless	4	4	A	8.5
Poor appetite	3	4	A	8.5
Can’t pay attention	3	4	A	8
Child made fun of because of snoring	5	5.5	C	6.5
Overall, how much of a problem has emotional distress been for your child during the past 4 weeks because of enlarged tonsils and adenoids?	6	5.5	A	9
**Activity limitations**	**1**	**1.5**	**A**	**9**
Playing	1	1	A	9.5
Participating excelling at sports	4	3	B	7
Doing things with friends/family	3	3.5	A	8
Attending school or day care	3	3	A	8.5
Overall, how much have your child’s activities been limited during the past 4 weeks because of enlarged tonsils and adenoids?	7	5	A	9
**Caregiver concerns**	**3**	**3**	**A**	**7.5**
Have you, as a caregiver, been worried, concerned, or inconvenienced because of your child’s snoring and difficulty breathing at night during the past 4 weeks.	7	5	A	8.5

**Table 2:** Analysis of internal consistency of the OSD-6 questionnaire.

OSD-6 Domain	No. of Items	% Floor Effect	% Ceiling Effect	Cronbach’s α
Physical suffering	1	—	20%	0.78
Sleep disturbance	1	—	20%	0.76
Speech or swallowing problems	1	15%	—	0.76
Emotional distress	1	—	—	0.78
Activity limitations	1	15%	—	0.78
Caregiver concerns	1	—	20%	0.76
Overall	6	**2%**	**2%**	**0.8**

**Table 3:** Concurrent validity of the OSD-6 questionnaire measured with Pearson correlation coefficient.

OSD-6 Domain	Physical Suffering	Sleep Disturbance	Speech or Swallowing Problems	Emotional Distress	Activity Limitations	Caregiver Concerns	Overall
**Physical suffering**	1	0.32	0.50	0.42	0.37	0.45	0.73
**Sleep disturbance**	0.32	1	0.34	0.23	0.24	0.77	0.64
**Speech or swallowing problems**	0.50	0.34	1	0.34	0.32	0.58	0.74
**Emotional distress**	0.42	0.23	0.34	1	0.55	0.30	0.68
**Activity limitations**	0.37	0.24	0.32	0.55	1	0.27	0.65
**Caregiver concerns**	0.45	0.77	0.57	0.30	0.27	1	0.78
**Overall**	0.73	0.64	0.74	0.68	0.65	0.78	1

**Table 4:** Predictive validity of OSD-6 in relation to Mallampati score and apnea-hypopnea index (AHI).

	No. of Children	Mean OSD Score	SD	Bonferroni ANOVA *p* Value
**Mallampati**				0.011
Class I	3	1.23	1.18	
Class II	27	3.4	1.23	
Class III	23	3.9	1.32	
Class IV	7	3.3	1.22	
**AHI level**				0.069
Normal	8	2.6	1.60	
3–5/h^−1^	7	2.76	1.56	
5–10/h^−1^	13	3.7	1.33	
>10/h^−1^	32	3.8	1.2	

ANOVA, analysis of variance; SD, standard deviation; AHI, apnea-hypopnea index.

**Table 5:** Analysis of test-retest repeatability.

OSD-6 Domain	ICC (95% CI)
Physical suffering	0.94 (0.92–0.96)
Sleep disturbance	0.97 (0.93–0.99)
Speech or swallowing problems	0.89 (0.85–0.94)
Emotional distress	0.85 (0.82–0.89)
Activity limitations	0.91 (0.87–0.95)
Caregiver concerns	0.79 (0.75–0.86)
Overall	0.92 (0.89–0.95)

CI, confidence interval; ICC, intraclass correlation coefficient.

**Table 6:** OSD-6 questionnaire sensitivity to change.

OSD-6 Domains	Mean Score before Treatment	Mean Score after Treatment	*p* Value
Physical suffering	4 (SD 2)	1 (SD 1)	0.001
Sleep disturbance	5 (SD 2)	1 (SD 2)	0.001
Speech or swallowing problems	3 (SD 2)	1 (SD 1)	0.001
Emotional distress	3 (SD 2)	1 (SD 1)	0.001
Activity limitations	2 (SD 2)	1 (SD 1)	0.001
Caregiver concerns	4 (SD 2)	1 (SD 2)	0.001
Overall	21 (SD 8)	6 (SD 6)	0.001

SD, standard deviation.

## Data Availability

The dataset supporting the conclusions of this article is available from the corresponding author, upon reasonable request.

## Supplementary Materials

The following supporting information can be downloaded at: https://www.mdpi.com/article/10.3390/jcm14051709/s1, Figure S1: Original version of OSD-6; Figure S2: Spanish version of OSD-6.

## Abbreviations

The following abbreviations are used in this manuscript:

OSA	Obstructive Sleep Apnea
HRQoL	Health-related quality of life
T14	Pediatric Throat Disorders Outcome Test
ICC	Intraclass correlation coefficient
AIDS	Acquired immunodeficiency syndrome
BMI	Body Mass Index
AHI	Apnea-Hypopnea Index
EMG	Electromyography
SD	Standard deviation
ANOVA	Analysis of variance
SEPAR	Spanish Society of Pulmonology and Thoracic Surgery
