# Supplementary material for: Cross-Cultural Adaptation of a Health-Related Quality-of-Life Questionnaire for Children with Obstructive Sleep Disorders: Spanish Version of the OSD-6
Source: J Clin Med. 2025 Mar 3;14(5):1709. doi: 10.3390/jcm14051709 (PMC11900155; doi:10.3390/jcm14051709)
Supplement: Supplementary file 1 [file jcm-14-01709-s001.zip › jcm-3486078-supplementary.pdf]

SUPPLEMENTARY MATERIAL

Impact of Obstructive Sleep Disorders on Child Quality of Life

Please help us understand the impact of enlarged tonsils and adenoids on your child's sleep-related quality of life. Please circle any symptom(s) that affect your child as well as the number that describes the severity of the problem for each question below.

Problem List:	Problem Scale (Circle Best Answer Below):
	None | Hardly at All | Somewhat | Moderate | Quite a Bit | Very Much | Couldn't Be Worse
**1. Physical Suffering:** For example: Sore throat, dry throat, nasal congestion, completely blocked nose, bedwetting, excessive daytime tiredness, failure to gain weight, bad breath. **Overall, how much has physical suffering been a problem for your child during the past 4 weeks because of enlarged tonsils and adenoids?**	0 1 2 3 4 5 6
**2. Sleep Disturbance:** For example: Snoring, choking/gasping for air, stopping breathing for a few seconds, restless sleep, difficult to awaken from sleep, chest caving in with breathing. **Overall, how much has sleep disturbance been a problem for your child during the past 4 weeks because of enlarged tonsils and adenoids?**	0 1 2 3 4 5 6
**3. Speech or Swallowing Problems:** For example: Difficulty swallowing certain foods, choking on foods, muffled speech, nasal sounding speech, poor pronunciation. **Overall, how much has speech or swallowing been a problem for your child during the past 4 weeks because of enlarged tonsils and adenoids?**	0 1 2 3 4 5 6
**4. Emotional Distress:** For example: Irritable, frustrated, sad, restless, poor appetite, can't pay attention, child made fun of because of snoring. **Overall, how much of a problem has emotional distress been for your child during the past 4 weeks because of enlarged tonsils and adenoids?**	0 1 2 3 4 5 6
**5. Activity Limitations:** For example: Playing, participating/excelling at sports, doing things with friends/family, attending school or day care. **Overall, how much have your child's activities been limited during the past 4 weeks because of enlarged tonsils and adenoids?**	0 1 2 3 4 5 6
**6. Caregiver Concerns:** **Have you, as a caregiver, been worried, concerned, or inconvenienced because of your child's snoring and difficulty breathing at night during the past 4 weeks?**	0 1 2 3 4 5 6

Figure S1. Original version of OSD-6.

OSD-6

Impacto de los trastornos respiratorios durante el sueño en la calidad de vida infantil

Por favor, ayúdenos a comprender el impacto de la hipertrofia adeno-amigdalar en la calidad de vida relacionada con la salud de su hijo/a. Por favor, marque cada uno de los síntomas que afecten a su niño/a, así como el número que describe la gravedad del problema para cada una de las siguientes preguntas:

Nunca
Casi nunca
Pocas Veces
Regularmente
Frecuentemente
Casi siempre
Siempre

Lista de problemas:

Escala de problemas (marque la mejor respuesta debajo)

1. Sufrimiento físico:

Por ejemplo: dolor de garganta, sequedad de garganta, congestión nasal, obstrucción nasal, orinarse en la cama, cansancio excesivo durante el día, falta de aumento de peso, mala respiración
Globalmente, ¿cuánto problema ha supuesto este sufrimiento físico para su niño/a durante las 4 últimas semanas debido a la hipertrofia adeno-amigdalar?

0 1 2 3 4 5 6

2. Alteración del sueño:

Por ejemplo: ronquido, ahogo/jadeo, parada de la respiración, sueño no reparador, dificultad para despertar del sueño, trabajo respiratorio.
Globalmente, ¿cuánto problema ha supuesto esta alteración del sueño para su niño/a durante las 4 últimas semanas debido a la hipertrofia adeno-amigdalar?

0 1 2 3 4 5 6

3. Problemas del habla o deglución:

Por ejemplo: dificultad para tragar ciertos alimentos, atragantarse con alimentos, voz dificultad en el habla, voz nasal, mala pronunciación.
Globalmente, ¿cuánto problema ha supuesto estos problemas del habla o deglución para su niño/a durante las 4 últimas semanas debido a la hipertrofia adeno-amigdalar?

0 1 2 3 4 5 6

H.U. Sant Joan d'Alacant Neumología
Versión española del cuestionario OSD-6 (calidad de vida en el SAHS infantil)

4. Estrés emocional:
Por ejemplo: irritable, frustrado, triste, inquieto, falta de apetito, falta de atención, burlas de otros niños/as debidas al ronquido.
Globalmente, ¿cuánto problema ha supuesto este estrés emocional para su niño/a durante las 4 últimas semanas debido a la hipertrofia adeno-amigdalar? 0 1 2 3 4 5 6

5. Limitaciones de la actividad:
Por ejemplo: jugar, participar/sobresalir en deportes, hacer cosas con amigos/familia, asistir a la escuela o guardería.
Globalmente, ¿cuánto problema han supuesto estas limitaciones de la activdad para su niño/a durante las 4 últimas semanas debido a la hipertrofia adeno-amigdalar? 0 1 2 3 4 5 6

6. Preocupaciones del cuidador
¿Usted como cuidador ha estado preocupado, molesto o incomodado debido al ronquido o la dificultad para respirar durante la noche de su niño/a durante las últimas 4 semanas? 0 1 2 3 4 5 6

Figure S2. Spanish version of OSD-6.
